# Counseling at all contacts for postpartum contraceptive use: can paper-based tools help community health workers improve continuity of care? A qualitative study from Ethiopia

**DOI:** 10.12688/gatesopenres.13071.2

**Published:** 2021-04-29

**Authors:** Muluneh Yigzaw Mossie, Anne Pfitzer, Yousra Yusuf, China Wondimu, Eva Bazant, Vaiddehi Bansal, Devon Mackenzie, Deborah Sitrin, Tsigue Pleah

**Affiliations:** 1 Jhpiego, Addis Ababa, Ethiopia; 2 Jhpiego, Washington DC, 1776 Massachusetts Avenue NW, Suite 300, USA; 3 Department of Population Family and Reproductive Health,, Johns Hopkins Bloomberg School of Public Health, Baltimore, MD 21205, USA; 4 Jhpiego, Baltimore, MD 21231, 1615 Thames St # 200, USA; 5 Jhpiego, Conakry, Immeuble Guinomar, 5ème étage, Guinea

**Keywords:** PPFP, tracking tools, community health workers, continuum of care, community health information systems

## Abstract

**Background: **Globally, there has been a resurgence of interest in postpartum family planning (PPFP) to advance reproductive health outcomes. Few programs have systematically utilized all contacts a woman and her baby have with the health system, from pregnancy through the first year postpartum, to promote PPFP. Nested into a larger study covering two districts, this study assessed the use, acceptability, and feasibility of tools for tracking women’s decision-making and use of PPFP in the community health system in Oromia region, Ethiopia. Community-level tracking tools included a modified Integrated Maternal and Child Health (IMCH) card with new PPFP content, and a newly developed tool for pregnant and postpartum women for use by Women Development Armies (WDAs). Proper completion of the tools was monitored during supervision visits.

**Methods: **In-depth interviews and focus group discussions were conducted with health officials, health extension workers, and volunteers. A total of 34 audio-files were transcribed and translated into English, double-coded using MAXQDA, and analyzed using a thematic approach.

**Results: **The results describe how HEWs used the modified IMCH card to track women’s decision making through the continuum of care, to assess pregnancy risk and to strengthen client-provider interaction. Supervision data demonstrated how well HEWs completed the modified IMCH card. The WDA tool was intended to promote PPFP and encourage multiple contacts with facilities from pregnancy to extended postpartum period. HEWs have reservations about the engagement of WDAs and their use of the WDA tool.

**Conclusions: **To conclude, the IMCH card improves counseling practices through the continuum of care and is acceptable and feasible to apply. Some elements have been incorporated into a revised national tool and can serve as example for other low-income countries with similar community health systems. Further study is warranted to determine how to engage WDAs in promoting PPFP.

## Introduction

Globally, there has been a resurgence of interest in postpartum family planning (PPFP) to boost overall use of family planning (FP) ( Shah *et al.*, 2015) and improve maternal ( Sridhar & Salcedo, 2017), newborn, and child health outcomes ( DaVanzo *et al.*, 2007; DaVanzo *et al.*, 2008). Despite global and national efforts, the level of contraceptive use in the post-partum period in Ethiopia is still low and the unmet need is high. Modern PPFP uptake at six months after delivery is 25% and coverage of PPFP counseling is 20% ( Zimmerman *et al.*, 2019). In countries such as Ethiopia, use of community health workers, such as HEWs and WDAs, and integration of FP discussions during key contacts may improve PPFP uptake.

Operationally, PPFP involves integrating FP discussion at key contacts a woman or her baby make with the health system, such as counseling for PPFP during antenatal care (ANC), at the time of delivery, in the immediate postpartum prior to discharge ( Pfitzer *et al.*, 2015), or during child visits such as immunization services ( Cooper *et al.*, 2015; Huntington & Aplogan, 1994; Ryman *et al.*, 2012; Vance *et al.*, 2014). The World Health Organization (WHO) developed programming strategies recommending systematic integration ( WHO, 2013), yet, ensuring that all providers consistently do so at each contact is challenging even with program support ( Cleland *et al.*, 2015).

Systematic integration involves provider action to discuss reproductive intentions and PPFP during each contact a woman or her baby have with the health system. Different strategies to influence provider actions or behavior show varying levels of effectiveness ( Rowe *et al.*, 2018). Within the context of behavior change programming, the emerging field of behavioral economics suggests that prompts or nudges can increase the performance of a desired behavior ( Matjasko *et al.*, 2016). A recent study in Ethiopia applied a behavioral economics approach to the problem of contraceptive discontinuation, using a pictorial checklist to remind providers to mention potential side effects to clients during counseling ( JSI Research & Training Institute Inc (JSI) & ideas42., 2018). With respect to PPFP, use of antenatal cards or stamps that record a woman’s method choice have been used in various countries ( Pfitzer *et al.*, 2015; Pleah *et al.*, 2016). Designing tools with prompts to remind or nudge providers to counsel women about FP at multiple contacts during ANC, childbirth, postnatal care, or immunization holds promise for changing provider behavior.

On the client side, intentions to use PPFP may be formed in the antenatal period ( Keogh *et al.*, 2015), but are acted upon in the postnatal period. Repeated counseling during ANC and combinations of antenatal and postnatal counseling lead to increased PPFP use ( Cleland *et al.*, 2015). Routine health management information systems do not typically facilitate continuity of care ( Akashi *et al.*, 2018), however, because data are dispersed across different health care units and facilities. This makes it challenging to link women’s decisions during antenatal counseling with postpartum contraceptive service delivery, especially if providers are unable to access information on services provided previously. Given the many contacts women can have during and after pregnancy at facility and community levels, it becomes difficult for providers to follow women over time and ensure that they are effectively counseled on PPFP, are able to decide about use, and receive their selected method.

In Ethiopia, the Federal Ministry of Health (FMOH) instituted a Health Extension Program ( Banteyerga, 2011; Karim *et al.*, 2013; Teklehaimanot & Teklehaimanot, 2013; Tilahun *et al.*, 2017), engaging Health Extension Workers (HEWs) to provide community health services. HEWs oversee a cadre of volunteers, sometimes called the “health development army” but referred to here as “women development army” (WDA) to support their work. HEWs are operational at health posts and track health data through Ethiopia’s Family Folder System ( Greene *et al.*, 2014; Sebastian & Lemma, 2010), which documents data related to each household and the individuals living there.

Part of the Family Folder system is the Integrated Maternal and Child Health (IMCH) card. HEWs use the card to document care provided to mother and child, from antenatal care through completion of immunizations and growth monitoring in the second year of the child’s life. WDA members encourage women in their communities to access maternal, newborn, child health, FP and nutrition services, though typically without any tools to standardize or structure their work ( Ashton *et al.*, 2015). In agreement with the FMOH, we modified the IMCH card to include prompts and facilitate tracking of PPFP choices and uptake over time. We also introduced a new WDA tool to promote referrals along the continuum of care, including PPFP use.

This paper presents an assessment of the use, acceptability, and feasibility of the modified IMCH card to remind HEWs to discuss and document PPFP counseling and women’s decision-making, and a new WDA tool to encourage referral of women for PPFP and other health services. The results of this study will add evidence on the use and benefits of tracking tools to enhance global efforts to improve systematic integration of PPFP counselling and women’s uptake through the continuum of care.

## Methods

### Study design and setting

The testing of new and modified tools for PPFP is part of a larger quasi-experimental study exploring how to maximize all contacts a woman has with the health system during pregnancy, childbirth, and the first year postpartum (ClinicalTrials.gov registration number NCT03585361, posted July 13, 2018). The study was conducted in Hetosa and Lode Hetosa districts in Arsi Zone, Oromia Region. In each district, one primary health care unit (PHCU) - a health center with five satellite health posts - was randomly assigned to an intervention arm or comparison arm. Since multiple health posts report to the same health center, randomizing individual health posts would risk contamination.

Women were enrolled in their 2 ^nd^ and 3 ^rd^ trimester of pregnancy in February–March 2017. Study staff obtained consent and conducted enrollment interviews to collect demographic information, birth history, contraceptive knowledge, contraceptive use prior to pregnancy, and intention to use contraception after birth. Women were re-interviewed in May 2018 to collect endline information on contacts with the health system during and after pregnancy, information received on family planning, use of contraception since delivery, and infant feeding and immunization. The result revealed that postpartum women in the intervention arm were more likely to adopt contraception compared to women in the comparison arm ( Sitrin *et al.*, 2020).

After one year of intervention, the study team interviewed health officials, HEWs, and WDAs for their experiences and perspectives on the acceptability and feasibility of PPFP tracking tools and prompts. This qualitative assessment was done in both intervention and comparison clusters.

### Sampling

The study districts were selected because of the absence of other large-scale FP programs. We aimed to study the perspectives of all stakeholders who had experiences with the PPFP tracking tools and prompts.

The purposive sample for qualitative in-depth interviews (IDIs) included two zonal and four district health officials; four heads of primary health care units, and 23 HEWs from 18 health posts (10 intervention and 8 comparison; 18 interviews total as HEWs were interviewed jointly in health posts where two were available). In addition, we held six focus group discussions (FGDs) with 70 WDAs. No participant selected for IDIs or FGDs declined to participate. Sample size aimed to achieve saturation of themes. Additionally, up to five IMCH cards were pulled at each health post during supervision visits, conducted during implementation, to check for correct completion of the tool. A total of 180 IMCH cards were reviewed.

### Conceptual framework

**Figure 1.**
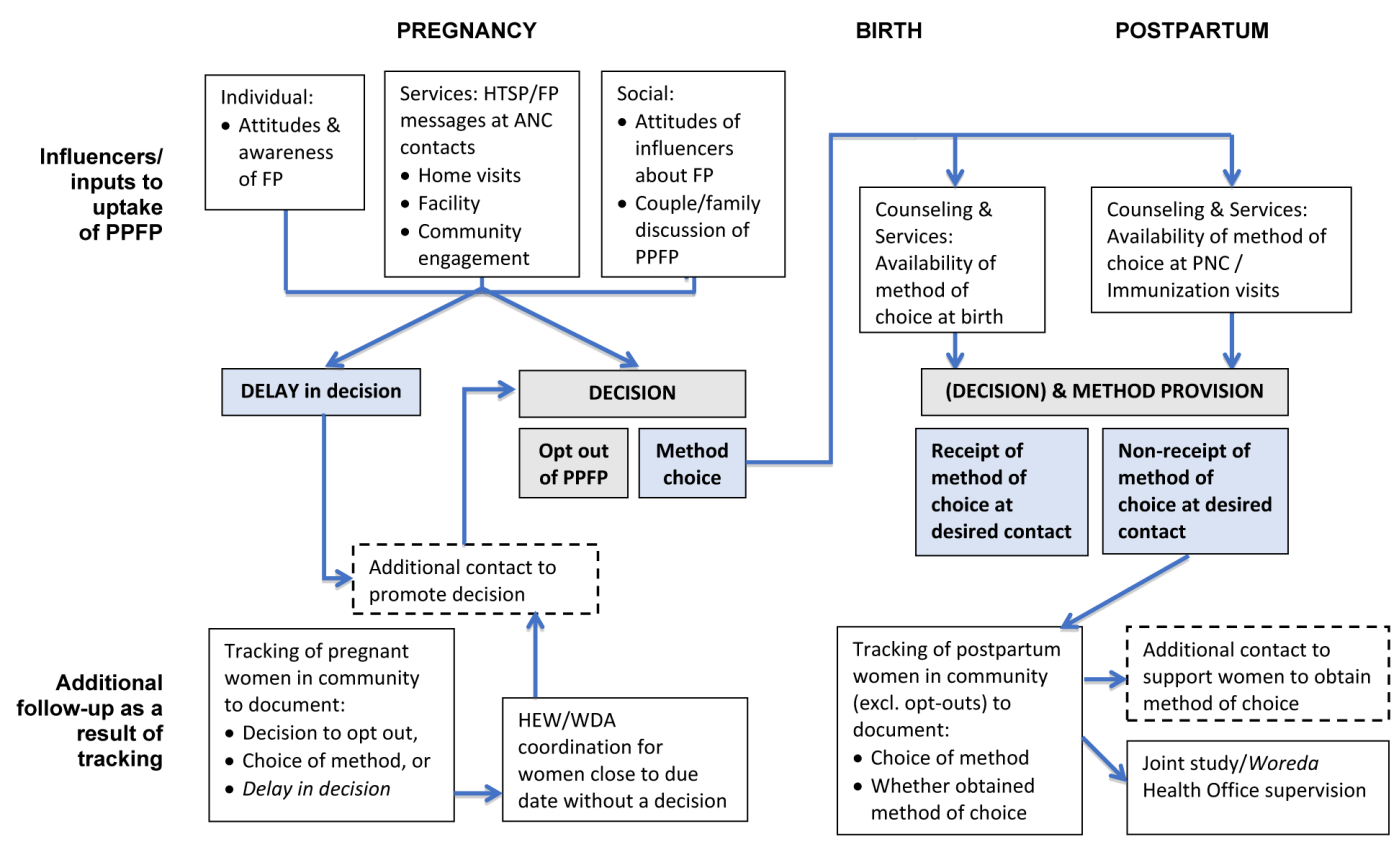
Conceptual framework showing influencers to increase uptake of postpartum family planning (PPFP).

A conceptual framework describing women’s decision-making and contraceptive use over the continuum of care guided the study design. This framework describes opportunities for two cadres of community health workers (CHWs), HEWs and WDAs, to influence women’s postpartum contraceptive use during service contacts at multiple levels of the health system ( Figure 1). This manuscript focuses on the analysis of HEW and WDA contacts with women and the tools used at the community health system level.

### Interventions

Interventions to strengthen PPFP counseling, provision, and documentation were implemented at all health centers in the study districts, including comparison PHCUs. Because the study was designed to assess the additional benefit of strengthening PPFP through the health extension program, only the intervention arm received the community intervention. The community intervention had three components: 1) training and supervision of HEWs on PPFP with a refresher on implant insertion, 2) giving HEWs and WDAs tools to track women’s PPFP preferences and pregnancy risk, and 3) having HEWs orient WDAs on PPFP messages and the WDA tool. The detail imputes for each study arm are outlined elsewhere ( Sitrin *et al.*, 2020).

The project made small modifications to the existing IMCH card to allow documentation of counseling, method preferences, and PPFP uptake at every contact during pregnancy and within one year after birth. The content of the IMCH card included PPFP counseling, method choice, and method adoption at each contact. The modified version of the IMCH card is available as *Extended data*; Mossie *et al*., 2019). In addition, prompts were added to ask women about return of menses, breastfeeding status, and complementary feeding at each growth monitoring visit to assess their return to fecundity and pregnancy risk. The FMOH provided approval to test the modified tools. We conducted regular visits to intervention health posts using a supervision tool (available as *Extended data*; Mossie *et al*., 2019) to gather observations on use of tools, capture responses to questions posed to HEWs, and extract PPFP data.

The WDA tool contained pictures of various contraceptive methods to help WDAs educate women on available options. It included boxes to check off a woman’s choice of PPFP method prior to delivery, along with boxes after birth to check breastfeeding status, use of FP, sexual activity, and if menses returned. The tool aimed to prompt WDAs to refer postpartum women to health posts if at risk of unintended pregnancy. The WDA tool is available as *Extended data*; Mossie *et al*., 2019).

### Study tools

We created semi-structured interview and FGD guides with questions on experiences of health care workers, HEWs, and WDA on their roles and responsibilities around PPFP and their use of PPFP tracking tools. The tools were prepared in English, translated into local language (Amharic and Afan Oromo), then back-translated. The interview guides were pilot tested in an area outside the study area with similar policy and cultural context.

### Data collection

Qualitative data were collected in May 2018. All FGD and in-depth interviews were audio-recorded and hand-written notes were kept after written informed consent was obtained from all participants. The FGDs lasted about 120 minutes and the IDIs were a maximum of 60 minutes long. The data collection was conducted by four research assistants. All research assistants had a postgraduate degree in public health and had received training for five days prior to the start of data collection. Supervisors oversaw field data collection.

### Data analysis

Research assistants who had participated in the data collection transcribed audio data in the original languages, Amharic and Afan Oromo. Two study members reviewed a sample of transcripts while listening to the audio data to confirm that there was congruence between the transcripts and audio data. The authors were public health and family planning program specialists and evaluators with experience in qualitative data analysis methods in health. Several authors, including the lead, are Ethiopian and two authors are from Oromia region and included men and women. After transcription, transcripts were translated to English for coding.

The study team developed initial codes based on the content of the transcripts and the study tools. The team then coded selected transcripts during a qualitative data coding workshop and adjusted the codes. The final codebook with definitions was uploaded to a qualitative data analysis software (MAXQDA 2018; Taguette is an open-access alternative) (Supplement 1). Each transcript was coded by two separate coders sequentially. After the second coding, coders discussed any discrepancies or disagreements to reach consensus on what codes should apply to various segments.

After transcripts were coded, the team analyzed the data using the ‘One Sheet of Paper’ technique ( Ziebland & McPherson, 2006). We reviewed segments for selected codes (including all related to tracking tools), sometimes grouped by type of respondent or study arm. We then summarized the various issues expressed on a single sheet of paper, including participant identifiers and links to specific coded segments (quotes), and developed written summaries for each code. Finally, we convened an analysis workshop, during which the investigators and analysis team reviewed each summary for similarity and outliers in views and opinions of study participants and identified emerging themes. Box 1 depicts the list of themes identified using the ‘One Sheet of Paper’ technique.

Box 1. **Themes relevant to tracking tools**

**The modified IMCH card**

1. Usefulness of the IMCH Card

- Serves as a behavioral ‘nudge’ for HEWs
- Serves as a tool to assess ‘risk of pregnancy’ in extended postpartum period
- Tracks women’s decision-making through the continuum of care
- Strengthens client-provider interaction and build trust between HEWs and clients

2. Completeness of the IMCH card

3. Acceptability and feasibility of the IMCH card

**The WDA tool**

1. Usefulness of the IMCH card

- Encourages referrals for PPFP and other health services
- Supports WDAs to provide client education and document services
- Helps WDAs and women understand connections between exclusive breastfeeding, complimentary feeding, and pregnancy risk

2. Acceptability and feasibility of the WDA tool

To support validity, preliminary findings were presented to select study participants – HEWs, health care workers, and health officials – to check that the results and interpretations agreed with their views and opinions

### Ethics

The study was approved by the JHSPH-IRB (IRB No: 7143). A support letter was obtained from the Ethiopian Ministry of Health to conduct the study. Oral consent was sought and received from all study participants. We did not seek written consent because of some participant’s low literacy level, concern about collecting paperwork with identifying information and the minimal risk associated with the study. Every participant received a copy of the consent form in their preferred language.

## Results

### Characteristics of study participants

The study enrolled 103 participants in both intervention and comparison sites, predominantly WDAs (70 participants in 6 FGDs) and HEWs (23 participants). The characteristics of study participants are presented in Table 1. The following sections explore how HEWs and WDAs used the tools introduced by the study, and the acceptability and feasibility of the modified IMCH card and WDA tool for tracking women’s decision-making and PPFP use.

**Table 1.** Characteristics of study participants.

HEW	Intervention (N=10)	Control (N=8)
Age (years)	28.3±4.1	27.8±6.1
Experience as HEW (years)	8.8±3.5	8±2.7
Married (yes, %)	100.0	87.5
Lives in kebele (yes, %)	90.0	62.5
**FGD (WDA)**	Intervention (N=44)	Control (N=26)
Age (years)	34.0±8.2	29.5±5.7
Education (no, %)	34.1	55.7
Occupation (Farmer, %)	95.5	100.0
Married (yes, %)	90.9	100.0

HEW: Health Extension Worker; FGD: focus group discussion; WDA: Women’s Development Army.

### The modified IMCH card

***How do HEWs use the modified IMCH card?***

***1. As a HEW behavior ‘nudge’***

The modified IMCH card serves as a “nudge” reminding HEWs to conduct PPFP counselling during each visit through the continuum of care. A HEW described how recording choice of PPFP method for pregnant women helps providers follow up with women after birth to offer the FP method she chose.

*“When a mother comes to us the first thing [we do] is we register … there is also a section which asks on counselling. If I counselled her, I do register it on this. If in case I forget to counsel, the card also reminds me to counsel her... This card helps me to record her [method of] choice during ANC and remind me [to provide it] ....” --HEW33, intervention*

***2. As a tool to assess ‘risk of pregnancy’***

HEWs thought that the modified IMCH card also helped to assess whether a woman is at risk of pregnancy, and thus may need contraceptives, based on months postpartum, breastfeeding practices, or return of menses. One HEW (HEW33, intervention) stated, it “ *helps me, opening a new way for counselling*” around risk of pregnancy, noting that in the previous card, “ *there was no such opportunity in the past to identify mothers [at risk of pregnancy]*”. Another HEW (HEW34, intervention) said: *“…for the mother, on her return if she sees her menses, we counsel her to start family planning”*. All HEWs appreciated the section on assessing pregnancy risk, though one (HEW38, intervention) talked about being confused on how to complete the pregnancy risk section, even though she strongly supported the idea.

***3. To track women’s decision-making through the continuum of care***

HEWs noted that they counsel their clients over several visits, and it can take repeated counseling before a woman is ready to adopt PPFP. All HEWs who participated in the study stated that the card helped them track and continuously follow up with their clients, until the woman makes a decision. One modification to the IMCH card was to record method choice during pregnancy, which allowed HEWs to refer to it during later visits, for example, during childbirth. However, women are not expected to stick to their original choices and may choose other methods if they change their mind during subsequent visits. Once they agreed to use contraceptives, HEWs provide the service and record the uptake on the IMCH card. Next, HEWs will regularly check the client if there are reports of complications with the method used. Two HEWs explained:

*“…this mother already chose [a method] during pregnancy and what she chose is with us. Now it’s to remind her to start the service. If she is willing, we provide her on the first day she comes … She can also change her first choice too. …. If she changes her idea after birth, we provide for the mother on her second choice. Therefore this [IMCH] card has a space to fill her choice. There is also a space where the method received [is] filled*. “—HEW38, intervention
*“…it will continue [to educate women] up to one-year post delivery. We will counsel [clients] without a sense of fatigue. We will counsel them to space between births and to take FP … if they agree to use, we will record that … and we will be grateful; next time, [we will assess] if any of them develop some complications. Apart from that we don’t say anything”*-- HEW40, intervention

***4. To strengthen client-provider interaction and build trust***

Many HEWs commented that the IMCH card strengthened interactions and built trust between themselves and their clients as they received more than one service at a time. Clients received FP counseling while primarily visiting the health care facilities for other services, such as child health care and other services during pregnancy and in the postpartum period. This was clearly narrated by one of the HEWs:

“ *It [the card] changes [the interaction between HEWs and clients] because the clients get more than one service [at a time]. When they come for their child care … they can get FP or get comprehensive service and this changes the interaction. Our interaction is strengthened.*” -- HEW 39, intervention

One HEW (33, intervention) commented that the modified IMCH card prompts reminders about breastfeeding and how exclusive breastfeeding or the lactational amenorrhea method (LAM) can be used to delay pregnancy: *“When we tell mothers the fact that breastfeeding the child properly will prevent pregnancy, they are very happy. That helped [HEWs] to establish better relationship with them [clients]”* (HEW33, intervention).

***Did HEWs properly complete the modified IMNCH card?***

As shown in Table 2, during each supervision visit, up to five IMCH cards were selected at each health post (180 total) to review data quality and assess PPFP services provided. HEWs recorded almost all women who received FP services. Very few women came to health posts for postnatal care; most of those that did were recorded as having chosen an FP method. Less than half of women had brought their babies for immunization at health posts; those that did were recorded as receiving counseling on FP.

**Table 2.** Completeness and quality of modified maternal, newborn and child health Cards (n = 180).

Data completeness and quality ^a^	%
HEW recorded if FP counselling done and outcome at each pregnancy visit	76.0
HEW recorded if FP counselling done and outcome at each postnatal visit	99.0
HEW recorded the pregnancy risk assessed at each growth monitoring visit	99.0
HEW correctly assessed pregnancy risk at each growth monitoring visit	98.0
**Outcomes**	
HEW recorded the FP method chosen during pregnancy	96.0
HEW recorded the FP method chosen during postnatal visit	99.0

^a^ Note that cards removed randomly may not have all sections completed, for example if the woman is still in pregnancy or early postpartum. Blank sections of the card were removed from the denominator in this table. FP: family planning; HEW: Health Extension Worker.

***How acceptable and feasible is the modified IMCH card?***

HEWs thought the modified IMCH card is feasible to use. The changes to the card helped HEWs to document all services delivered to their clients over time. It also allowed them to document which clients received PPFP counselling and to record method choice. Indeed, HEWs thought that the card has all the information they needed to provide to women during counselling sessions:

*“…the previous card did not have a section on counselling for mothers; whether we provide counselling or not… there was no space to record that... And also, in the past, we used to send mothers home without registering their preferred FP methods after the counselling; but now, we do actually register the FP [method] that they have chosen. This helps us to easily implement later. So in my view all the section in the card are very comfortable or easy to use.”* --HEW33, intervention

Moreover, the contents of the IMCH card were organized in a way that facilitated easy completion. Unlike tools used in the past, HEWs documented all interventions in the modified IMCH card using a code, so it did not take much time to write each of the services provided to clients. As one HEW described:

*“What makes this card different is, starting from ANC, it contains PNC and FP. You don’t need to write on it, it has code. FP has its own code. What she needs will be selected from the code. Compared with other cards, we are so comfortable with this one.*” -- HEW 36, intervention

One of the HEWs noted that the program will be sustained even without external assistance as the card is very helpful. She further noted that, as the copy of the card is with them, they will continue to use it after the project is phased out.

*“… even if the NGO might not be successful and the program failed, as the [IMCH] card is with us, we will continue to use because it is very helpful… the card is very good”.* – HEW 40, intervention

Health officials in the study sites also appreciated the modified card’s simplicity to use. Two officials (LHET10, ZON11) specifically highlighted that the card is integrated, and hence increased its acceptance and sustainability in the healthcare system. Another official characterized the card as ‘clear and productive’ and expressed interest to use it widely:

“ *According to the Federal Ministry of Health, there is no separate card for postpartum family planning. Yes, I have seen the one that prepared by [the study team]. It was prepared in briefest and clearest form. If you want to use that card, you could easily identify all things without any effort. Mother’s criteria are clearly outlined in the card. If she is using family planning or if she could be at risk [of pregnancy], it is identified in question form. Working on all things all in all could be good; but according to our woreda … the work limited to health posts should be expanded to other areas. If all do similar things, that work would be profitable..*.” --Health Official 7

### The WDA tool

***How do WDAs use the WDA tool?***

***1. To encourage referrals for PPFP and other health services***

FGD participants noted that the WDA tool is helpful to encourage use of services, with pictorials for ANC, childbirth, and immunization. FGD participants from intervention sites noted that after educating women on FP, they often refer them to health care facilities to receive their contraceptive choice. They also said that WDAs accompany their clients while traveling to health care facilities to receive contraceptives. An FGD participant explained:

“ *I gather pregnant mothers and counsel them on what we learn, then I sign on the [WDA] card to confirm the women received FP education …: I fill on the cards that I provided advice for them [women]. We go to the health facility together..*.” --FGD 03, P#3, intervention

However, WDAs in one of the comparison areas also said that they advise FP for their clients after birth and refer to health posts to receive their choice, even without the WDA tool. One of the participants, for example stated: *“We advise husbands to take his wife to health post and use family planning right before sexual intercourse.” --*FGD 06, P#12, comparison.

***2. Support WDAs to provide client education and document PPFP services***

Some FGD discussions briefly touched on the helpfulness of the WDA tool to provide FP through the continuum of care. WDAs recounted how they documented their work and educated pregnant women according to the guidelines on the WDA tool. As one stated:

“… *this tool supports me: [when] I meet a pregnant woman, [I] ask her how many months pregnant [she is]. When she told me it’s about a month, I put a mark in the box in front of it, then I put a mark on the second and third, then till she reached nine months, I put a mark there. When she completed nine months and give birth … then I proceed to breastfeeding … on breastfeeding area up to six months it’s here, then it has up to nine months. In the ninth month box I could look at how to feed and how to receive vaccination and put a mark on it. Then after … how she use family planning, the different methods: injectable, pills, ‘Loop’ [IUD] are available. When she says I need injectable … when she says I need ‘Loop’, I advise her to visit a health professional …..” –* FGD 04, P#3, intervention

FGD participants liked having something to show women; as one declared (FGD 04, P#6, Intervention): “ *This card is better, it helps us to show and educate rather than oral talk”*.

Despite the benefits mentioned above, some FGD participants showed a lack of knowledge and reported giving inaccurate advice to women or expressing uncertainty over how to speak with them about particular topics. The tool may thus confer them status that is not warranted by their level of expertise. An FGD participant narrated her guidance:

*“Many people asked me what type of problem [is] linked with the method inserted in the uterus and I told them in the future it might lead to sterility… I don’t know in detail, but I heard there was an individual that use 7-year method [IUD], then after 7 years she became sterile. I have no such experience, but for most pregnant women what I advise is not to think about other methods, take injectables every three months, and if you fear injectables take pills.”* –FGD 03, P#9, intervention

***3. To Help WDA and women understand connections between exclusive breastfeeding, complimentary feeding, and pregnancy risk***

While the WDA tool was not expected to provide comprehensive nutrition guidance, the inclusion and emphasis on LAM touches on feeding practices and behaviors. For example, one FGD participant stated:

“ *I advise them [women] how they can space their children, how to breastfeed their children, about breastfeed their child until six months, how they can give additional food to their children after six months*.” --FGD 01, P#3, intervention

A WDA in the comparison group also frequently mentioned giving nutrition advice. The discussion reflects exposure of WDAs in that ward ( *kebele*) to nutrition activities. However, in those focus groups, nutrition and FP advice is mentioned more in a list of activities, without linkages between them, along with the importance of institutional delivery and postnatal care.

*“First, she [a woman] has to give birth at health facility. After she gives birth and returned home, she has to go to facility to check up her newborn and herself. When the newborn is checked and found to have malnutrition, the facility will provide balanced food. I will advise such things. After she returned home she has to get adequate food. When she get adequate food, her child will also be stronger. Then she has to start contraceptives…. We will check the hygiene of herself and her child. She has to eat balanced diet. After her child become 6 month, she has to start additional balanced diet. And also she has to continue breastfeeding until her child is two years old. ….”* --FGD 06, P#2, comparison

***How acceptable and feasible is the WDA tool?***

HEWs have mixed views about WDAs’ understanding of PPFP concepts and their ability to effectively use the WDA tool. Two HEWs (HEW 40 and 41) talked about the need to help the WDAs fill out their tools, as most have no education. In contrast, some participants stated that WDAs can properly use the tool. One (HEW 33) noted that having pictorial cards was helpful to the WDA, though she observed that some WDAs initially removed the cards from the binder ring and gave them away instead of keeping the cards within the ring. However, she reported that card utilization improved over time. Another HEW (HEW 39) noted that WDAs’ use of the card was inconsistent, with some properly using them and others not. One HEW (HEW 36) felt that WDAs were using the card only because HEWs forced them, or because of the study team’s influence. This same HEW felt that WDAs are limited in the FP information they can share with women. Another HEW (HEW 34) reported that the acceptance of WDAs by their peers is low and thus limited their ability to use the tool. She stated the mothers would tell the WDAs, *“you are simply boosting yourselves, you don’t know anything.”*

## Discussion

We examined the use, feasibility, and acceptability of tracking tools at the community health level to improve counseling and facilitate decision-making and uptake of contraceptives during pregnancy and in the extended postpartum period. Both HEWs and health officials thought that the modified IMCH card is acceptable and feasible to use. Review of a sample of IMCH cards found that HEWs properly completed the modified IMCH card, reinforcing their feedback that the card is easy to use. In contrast, there are mixed results on the utility and feasibility of the PPFP tool for WDAs. While most WDAs felt positively about the tool, an overwhelming number of HEWs argued that the WDAs’ literacy posed challenges in understanding how to properly use the tool. HEWs further noted that providing training to WDAs is difficult, albeit with a few exceptions for highly motivated volunteers.

Past studies have indicated that community-based data can support community health workers’ decision-making related to health and healthcare ( de la Torre & Unfried, 2014; Jeremie *et al.*, 2014; Karim *et al.*, 2018). Studies report that tracking tools and prompts can reduce treatment dropouts, decrease missed opportunities, and increase uptake of community-based interventions ( Chewicha & Azim, 2013; MEASURE Evaluation, 2016), for example, in the treatment and follow-up of tuberculosis ( Fraser *et al.*, 2007). However, tools for community health systems should be designed in a language understandable by CHWs ( Chewicha & Azim, 2013; Jeremie *et al.*, 2014). Chewicha & Azim (2013) commented on the importance of ensuring that the tools given to CHWs are easy to understand. Others have reported that the use of tracking tools within community health systems is feasible, acceptable, and can strengthen CHWs performance ( Kaseje *et al.*, 2010; Sabitu *et al.*, 2004; Singh *et al.*, 1997).

This study showed that WDAs are playing key roles to promote PPFP and refer clients to facilities for various maternal, newborn, and child health services as well as FP services. We also find persistent misconceptions among some WDAs related to contraceptives. For example, one FGD participant noted that she encourages women not to take IUDs because she believes that IUD causes sterility. Other authors have found that CHWs sometimes lack knowledge on how to effectively perform their responsibilities unless they are properly trained, and their understanding of new concepts closely monitored ( Malaviya *et al.*, 2013; Martinez *et al.*, 2008; Onwuhafua *et al.*, 2005). The issues rose on the skills of HEW/WDA in this study warrant further investigation.

One approach is to maintain the positive contributions of WDAs to identify pregnant women and notify HEWs. This is especially useful in remote parts of the *kebeles*. Rather than relying on WDAs to provide the level of information currently captured in the study’s WDA tool, HEWs could work with WDAs to gather groups of women for education sessions that the WDA themselves could participate in as “helpers,” thus progressively enhancing their own knowledge. HEWs noted that select WDAs exhibited greater interest in and motivation to work on PPFP. HEWs could perhaps identify such individuals and selectively decide whether to orient them on the use of the cards.

Ethiopia has created a unique community health information system, using a family folder for every household in the country ( Damtew & Moges, 2013). However, the family folder is a recent phenomenon in Ethiopia, and its potential may not be fully realized ( Greene *et al.*, 2014). Based on preliminary experience from this study, the FMOH approved revisions to include PPFP counseling, method choice, and uptake in the pregnancy, postnatal, and immunization sections of the new IMCH card. However, the study’s innovation of prompting HEWs to assess various LAM criteria, re-labeled as pregnancy risk, was not incorporated into revisions to national tools. HEWs expressed appreciation for this component as it introduced “a new way of counseling” women on the possibility of a pregnancy. This is encouraging for resolving the persistent problem of timely transition from LAM to other modern contraception in programs that have sought to promote LAM ( Cooper *et al.*, 2014; Cooper *et al.*, 2016; Kouyaté *et al.*, 2015). For Ethiopia, additional changes to the IMCH cards are unlikely in the short term given the recent revisions of national tools. Thus, prototyping alternative means to arrive at the same result can be explored in the short term, such as separate job aids, or stickers to add to the IMCH card or folder.

A recent systematic review of evidence by Rowe *et al.* (2018) on interventions to improve provider practices suggested that job aids alone will not change provider practices. Our study included a combination of tracking tools, with nudges and supervision to observe and encourage PPFP counseling. The effectiveness of the interventions on PPFP uptake published separately and revealed that postpartum women in the intervention arm were more likely to adopt contraception compared to women in the comparison arm ( Sitrin *et al.*, 2020). Based on the results of the effectiveness and qualitative studies, the IMCH card is feasible to use and we recommend use of prompts to encourage systematic integration of PPFP counseling and services

Quantitative data on PPFP uptake will be published separately. Nevertheless, we feel the qualitative data on the IMCH card are sufficient to recommend use of prompts to encourage systematic integration of PPFP counseling and services. Furthermore, we recommend that reforms of information systems should strive to make documented method choice in pregnancy or early postpartum available to providers seeing women later in the postpartum period, when fecundity may be returning due to lapses in exclusive breastfeeding or menses return. However, as Rowe *et al.* (2018) prescribe, multiple strategies will enhance providers’ systematic integration of PPFP.

In 2016, the Federal Ministry of Health developed an information road map for the health sector, dubbed ‘the information revolution road map’ ( FMOH, 2016). The road map boldly outlines how to improve the routine health information system to facilitate decision-making at various levels. This study contributes a case study about how nudges to prompt health care workers to review client records of previous visits can improve continuity of care. HEWs found that the tool modifications tested in this study led them to easily refer to women’s decision-making around PPFP noted in prior sections of the card and to gauge what additional services she may want during that contact. While our focus centered on PPFP, there are likely other potential applications for clinical care, particularly for conditions monitored over time. We can draw lessons from the work of behavioral economics to inspire further small process improvements, such as PPFP counseling checks in each visit in this study, to improve provider compliance with clinical guidelines and protocols ( Ashton *et al.*, 2015). Data use on the spot to enable swift decision-making improves client-centeredness of care and service quality.

As countries move their health information systems to the digital realm, nudges or prompts can be built into such platforms. Already, there are groups developing biometrics solutions that can help ensure that digital tools can strengthen continuity of care from one health contact to another ( Storisteanu *et al.*, 2015). Our study sought to track PPFP through the continuum of care using paper-based records. Transforming such paper-based records to digital health records has potential to further improve service integration. Exploring such applications can easily incorporate items related to PPFP decision-making and follow up for timely adoption of a contraceptive prior to postpartum fecundity return.

### Strengths and limitations of the study

This study included the viewpoints of various health system stakeholders with different levels of expertise. Information was sought from health officials, health service providers, and community health workers. This study used rigorous analysis techniques to capture different views and opinions of study participants. There may have been courtesy bias where study participants avoid mentioning limitations of tracking tools. Instead, people may magnify positive comments and remain reserved about negative feelings. The weak functionality of the WDA program is another limitation of this study. In some areas, the WDAs are not actively working and may not use the tool. In such a situation, it would be difficult to judge whether the tool is acceptable and feasible. Lastly, the study did not assess whether the effects of modifying the IMCH card are long-lasting. There are concerns that behavioral nudges may not continue to generate the desired behaviors over time ( Marteau *et al.*, 2011; Matjasko *et al.*, 2016).

## Conclusion

The study revealed that the modified IMCH card is used as a behavioral nudge, tracks women’s decision-making through the continuum of care, and supports HEWs to identify pregnancy risk. The card improves the ability of HEWs to provide integrated FP services during pregnancy and in the extended postpartum period. The Ethiopian FMOH plans to scale a modified IMCH card. The use of integrated tools with specific PPFP nudges can also be replicated in other countries where frontline workers interact with women along the continuum of care. As countries develop digital health information systems, there will be renewed opportunities to build in prompts for providers to initiate conversations about PPFP.

Although WDAs witnessed the benefits of the WDA tool to facilitate client referral, many HEWs expressed reservations on the applicability of the tool. Discussions with HEWs suggest that WDAs struggled to understand some of the components of the WDA tool, particularly in relation to postpartum return to fecundity and risk of pregnancy prior to menses return. Some WDAs were not effective advocates for PPFP, either due to inactivity or misunderstandings about contraceptive methods. In the Ethiopian context, there needs to be further exploration of effective strategies to promote PPFP as a social norm.

## Data availability

### Underlying data

This article reports on the qualitative study interviews, conducted in a small geographic area, with a limited set of facilities. Investigators are concerned that de-identification of interview and focus group discussion transcripts to completely exclude deductive disclosure of respondent identities by individuals from the study area is not possible. In consultation with the JHU School of Public Health IRB, the authors have decided not to make the data generated accessible for sharing beyond the data contained within the article, except by request of researchers to Jhpiego’s Open Data help team at opendatahelp@jhpiego.org and upon negotiation of a Data Use Agreement that ensures reasonable protection of research subjects.

### Extended data

Open Science Framework: Counseling at All Contacts for Postpartum Contraceptive Use: Can Paper-based Tools Help Community Health Workers Improve Continuity of Care? A Qualitative Study from Ethiopia. https://doi.org/10.17605/OSF.IO/9FZYJ ( Mossie *et al*., 2019).

This project contains the following extended data:

- Modified_IMCH_Card yigzaw *et al.* (Modified Integrated Maternal and Child Care Card used for data collection in this study)
- Supervision_tool Yigzaw *et al.* (supervision tool used in this study)
- WDA Tool Yigzaw *et al.* (WDA tool used in this study)

### Reporting guidelines

Open Science Framework: COREQ checklist for ‘Counseling at all contacts for postpartum contraceptive use: can paper-based tools help community health workers improve continuity of care? a qualitative study from Ethiopia’. https://doi.org/10.17605/OSF.IO/9FZYJ ( Mossie *et al*., 2019).

Data are available under the terms of the Creative Commons Zero "No rights reserved" data waiver (CC0 1.0 Public domain dedication).
