# Supplementary material for: Counseling at all contacts for postpartum contraceptive use: can paper-based tools help community health workers improve continuity of care? A qualitative study from Ethiopia
Source: Gates Open Res. 2021 Apr 29;3:1652. Originally published 2019 Oct 30. [Version 2] doi: 10.12688/gatesopenres.13071.2 (PMC8094216; doi:10.12688/gatesopenres.13071.2)
Supplement: Peer review history

# Peer review: Reviewer response for version 2

**DOI:** 10.21956/gatesopenres.14483.r30625

**Published:** 2021-05-07

**Authors:** Nguyen Toan Tran

**Affiliations:** 1 Australian Centre for Public and Population Health Research, Faculty of Health, University of Technology Sydney, Sydney, Australia; 2 Faculty of Medicine, University of Geneva, Geneva, Switzerland

I have reviewed and am satisfied with the amended version and responses from authors.

Is the work clearly and accurately presented and does it cite the current literature?

Yes

If applicable, is the statistical analysis and its interpretation appropriate?

Not applicable

Are all the source data underlying the results available to ensure full reproducibility?

Yes

Is the study design appropriate and is the work technically sound?

Yes

Are the conclusions drawn adequately supported by the results?

Partly

Are sufficient details of methods and analysis provided to allow replication by others?

Partly

Reviewer Expertise:

Sexual and reproductive health and rights in humanitarian and development settings (clinical, capacity development, policies and programs).

I confirm that I have read this submission and believe that I have an appropriate level of expertise to confirm that it is of an acceptable scientific standard.

# Peer review: Reviewer response for version 2

**DOI:** 10.21956/gatesopenres.14483.r30623

**Published:** 2021-04-30

**Authors:** Alemayehu Hailu

**Affiliations:** 1 Department of Preventive Medicine, School of Public Health, Addis Ababa University, Addis Ababa, Ethiopia

All my comments are addressed.

Is the work clearly and accurately presented and does it cite the current literature?

Yes

If applicable, is the statistical analysis and its interpretation appropriate?

Not applicable

Are all the source data underlying the results available to ensure full reproducibility?

Yes

Is the study design appropriate and is the work technically sound?

Yes

Are the conclusions drawn adequately supported by the results?

Yes

Are sufficient details of methods and analysis provided to allow replication by others?

Yes

Reviewer Expertise:

Health Economist, Health systems research, Health policy, Public health, Health benefits package design.

I confirm that I have read this submission and believe that I have an appropriate level of expertise to confirm that it is of an acceptable scientific standard.

# Peer review: Reviewer response for version 1

**DOI:** 10.21956/gatesopenres.14207.r30043

**Published:** 2021-01-07

**Authors:** Alison L. Drake

**Affiliations:** 1 Department of Global Health, University of Washington, Seattle, WA, USA

During pregnancy and postpartum, there are opportunities to discuss FP while women are engaged in health care. Thus this time period is strategic to help promote birth spacing and limiting. In this study, healthcare extension workers were tasked with using a MCH care to track postpartum FP use over time and to use a new tool to promote referrals in Ethiopia. The authors assess uptake, acceptability, and feasibility of the card that is designed to remind HEWs to discuss PPFP and use the new tool. The study was nested within an ongoing RCT, with qualitative interviews of health care workers in intervention and control groups. A card with boxes to fill out to document FP counseling, uptake, and referral is a plausible approach to increase coverage of these services. Overall, the integrated IMCH card was very acceptable and easy to use - and facilitated documentation and delivery of FP counseling/services in pregnancy and postnatally.

 The authors should describe the parent RCT purpose/intervention. This should include the unit of randomization (was it clusters, who was randomized - clinics/health care workers?) to provide context. The difference between the intervention arm and community intervention is not clear. Also, what the control arm consisted of should also be presented. This has implications for generalizability of findings which cannot fully be assessed.

 Can more details be provided on the validity analysis? How were participants selected, how many, and how were results checked?

 Was there consideration of assessing risk of pregnancy before menses resume as there is risk in the 2 weeks before menses resume as well? 

 The authors should address the limitation of the skills/training of HEW/WDA. As noted in one FGD, there were myths that HEW were perpetuating that cannot be overcome with the IMCH or tool. Also, acknowledge the tool does not assist with quality of counseling. This example is used in the discussion, but there was no accompanying discussion about how to overcome myths/quality of counseling.

Is the work clearly and accurately presented and does it cite the current literature?

Yes

If applicable, is the statistical analysis and its interpretation appropriate?

Yes

Are all the source data underlying the results available to ensure full reproducibility?

No

Is the study design appropriate and is the work technically sound?

Yes

Are the conclusions drawn adequately supported by the results?

Yes

Are sufficient details of methods and analysis provided to allow replication by others?

Partly

Reviewer Expertise:

Epidemiology and reproductive health.

I confirm that I have read this submission and believe that I have an appropriate level of expertise to confirm that it is of an acceptable scientific standard, however I have significant reservations, as outlined above.

# Response

**Published:** 2021-04-04

**Authors:** Muluneh Yigzaw Mossie

1) During pregnancy and postpartum, there are opportunities to discuss FP while women are engaged in health care. Thus this time period is strategic to help promote birth spacing and limiting. In this study, healthcare extension workers were tasked with using a MCH care to track postpartum FP use over time and to use a new tool to promote referrals in Ethiopia. The authors assess uptake, acceptability, and feasibility of the card that is designed to remind HEWs to discuss PPFP and use the new tool. The study was nested within an ongoing RCT, with qualitative interviews of health care workers in intervention and control groups. A card with boxes to fill out to document FP counseling, uptake, and referral is a plausible approach to increase coverage of these services. Overall, the integrated IMCH card was very acceptable and easy to use - and facilitated documentation and delivery of FP counseling/services in pregnancy and postnatally.

 The authors should describe the parent RCT purpose/intervention. This should include the unit of randomization (was it clusters, who was randomized - clinics/health care workers?) to provide context. The difference between the intervention arm and community intervention is not clear. Also, what the control arm consisted of should also be presented. This has implications for generalizability of findings which cannot fully be assessed.

*Response:* Thank you for your comment. The larger study was a quasi-experimental study. The intervention and compression arm have been outlined in the methods section under the subtitles “Study design and setting “and “Intervention”.

 2) Can more details be provided on the validity analysis? How were participants selected, how many, and how were results checked?

*Response:* Thank you for your comment. The sampling process and the sample size are described at the methods section under the sub title “Sampling”. The result validation process is described in the data analysis section as follows:

 “To support validity, preliminary findings were presented to select study participants – HEWs, health care workers, and health officials – to check that the results and interpretations agreed with their views and opinions”.

 3) Was there consideration of assessing risk of pregnancy before menses resume as there is risk in the 2 weeks before menses resume as well?

*Response:* Thank you for your comment. Assessment of risk of pregnancy is part of the standard of care of family planning services for all clients in Ethiopia. In this study, we were assessing whether health extension workers were assessing the risk of pregnancy. The records were assessed and the results are shown in Table 2.   

 4) The authors should address the limitation of the skills/training of HEW/WDA. As noted in one FGD, there were myths that HEW were perpetuating that cannot be overcome with the IMCH or tool. Also, acknowledge the tool does not assist with quality of counseling. This example is used in the discussion, but there was no accompanying discussion about how to overcome myths/quality of counseling.

*Response:* Thank you for your comment. Unfortunately, the purpose of the study was to assess the uptake, acceptability, and feasibility of HEW/WDA’s *use of tracking tools*. We found from record review that health extension workers can properly fill the tools. However, some key informants raised the limitation of the skills/training of HEW/WDA. We discussed this at the third paragraph of the discussion section. At the same time, despite the reports that community health workers lack skills, there were also multiple reports from respondents that WDAs are playing key roles in promoting PPFP.  At the third paragraph, we added the following sentence to note our stand on skills of HEW/WDA.

*“*The issues rose on the skills of HEW/WDA in this study warrant further investigation. “

# Peer review: Reviewer response for version 1

**DOI:** 10.21956/gatesopenres.14207.r30040

**Published:** 2020-12-15

**Authors:** Nguyen Toan Tran

**Affiliations:** 1 Australian Centre for Public and Population Health Research, Faculty of Health, University of Technology Sydney, Sydney, Australia; 2 Faculty of Medicine, University of Geneva, Geneva, Switzerland

Many thanks for the immense opportunity to review this nested (and mostly – please see note below) qualitative study assessing the use, acceptability, and feasibility of tracking tools related to PPFP decision-making and use. I have a few comments for your consideration:

**Abstract**

 1. Results: please substantiate the major themes that emerged from your analysis (use, acceptability, feasibility…) and provide salient examples (e.g., what were the “reservations”; what is “how well HEW completed…”)

 2. Conclusions: please consider phrasing your conclusions in a more tentative way if this is a qualitative study stricto sensu (but it does not appear to be so – see comment under Methods).

**The Introduction**

 3. Please consider providing a brief summary of data on contraceptive use/PPFP in Ethiopia with levels of use, unmet needs, challenges and opportunities.

 4. Please describe the format and core contents of the WDA tool and IMCH card.

 5. Are the results of the main study available? Can you mention any preliminary results/remarkable findings? It would be helpful to interpret the results of this nested study in the context of the larger study.  

**Materials and Methods**

 6. The description of the qualitative methods is comprehensive and the framework much appreciated.  

 7. As the results also report quantitative data (Table 2), please add information on how you collected the data and reflect this mixed-method approach in the title, abstract and manuscript.

**Results**

 8. See commentary above on Table 2, which comes as a quantitative surprise.

**Discussion** **and Conclusion**

 9. Without data on effectiveness from the main study, the reader may find it difficult to support the following statement: “Quantitative data on PPFP uptake will be published separately. Nevertheless, we feel the qualitative data on the IMCH card are sufficient to recommend use of prompts to encourage systematic integration of PPFP counseling and services.”

 10. In relation to the previous comment, no doubt about the usefulness of reminders to systematically address and avoid missed opportunities in relation to PPFP (and other health areas). Could you provide more information and discussion on the time requirement and potential trade-offs for health workers to do so?

Is the work clearly and accurately presented and does it cite the current literature?

Yes

If applicable, is the statistical analysis and its interpretation appropriate?

Not applicable

Are all the source data underlying the results available to ensure full reproducibility?

Yes

Is the study design appropriate and is the work technically sound?

Yes

Are the conclusions drawn adequately supported by the results?

Partly

Are sufficient details of methods and analysis provided to allow replication by others?

Partly

Reviewer Expertise:

Sexual and reproductive health and rights in humanitarian and development settings (clinical, capacity development, policies and programs).

I confirm that I have read this submission and believe that I have an appropriate level of expertise to confirm that it is of an acceptable scientific standard, however I have significant reservations, as outlined above.

# Response

**Published:** 2021-04-04

**Authors:** Muluneh Yigzaw Mossie

**Abstract**

 1. Results: please substantiate the major themes that emerged from your analysis (use, acceptability, feasibility…) and provide salient examples (e.g., what were the “reservations”; what is “how well HEW completed…”)

*Response:* Thank you. The following is the revised result section of the abstract and is inserted in the second version of the manuscript:

 “HEWs used the modified IMCH card as a “nudge” reminding them to conduct PPFP counseling, to track women’s decision making through the continuum of care, to assess pregnancy risk and to strengthen client-provider interaction. Supervision data demonstrated how well HEWs completed the modified IMCH card. The modified IMCH card is simple to use, and this increased its acceptance and sustainability in the healthcare system. Unlike tools used in the past, HEWs documented all interventions in the modified IMCH card using a code, so it did not take much time to record each service provided to clients. HEWs have reservations about the engagement of WDAs and their use of the WDA tool.”

 2. Conclusions: please consider phrasing your conclusions in a more tentative way if this is a qualitative study stricto sensu (but it does not appear to be so – see comment under Methods).

*Response:* Thank you. The following is the revised version of the conclusion and is inserted in the second version of the manuscript:

 “The IMCH card improves counseling practices through the continuum of care and is acceptable and feasible to apply. Further study is warranted to determine how to engage WDAs in promoting PPFP.”

**The Introduction**

 3. Please consider providing a brief summary of data on contraceptive use/PPFP in Ethiopia with levels of use, unmet needs, challenges and opportunities.

*Response:* Thank you. The following is inserted in the first paragraph:

 “Despite global and national efforts, the level of contraceptive use in the post-partum period in Ethiopia is still low and the unmet need is high. Modern PPFP uptake at six months after delivery is 25% and coverage of PPFP counseling is 20% (Zimmerman 2019). In countries such as Ethiopia, use of community health workers, such as HEWs and WDAs, and integration of FP discussions during key contacts may improve PPFP uptake. “

 4. Please describe the format and core contents of the WDA tool and IMCH card.

*Response:* Thank you. The contents of the modified MCH card and the WDA card are outlined in the methods section under the subtitle “Intervention”. In the current version, the following has been inserted in the same paragraph:

 “The content of the IMCH card included PPFP counseling, method choice, and method adoption at each contact.”

 5. Are the results of the main study available? Can you mention any preliminary results/remarkable findings? It would be helpful to interpret the results of this nested study in the context of the larger study.  

*Response:* Thank you. The following is inserted in the second paragraph of the methods section:

 “Women were enrolled in their 2nd and 3rd trimester of pregnancy in February–March 2017. Study staff obtained consent and conducted enrollment interviews to collect demographic information, birth history, contraceptive knowledge, contraceptive use prior to pregnancy, and intention to use contraception after birth. Women were re-interviewed in May 2018 to collect endline information on contacts with the health system during and after pregnancy, information received on family planning, use of contraception since delivery, and infant feeding and immunization. The result revealed that postpartum women in the intervention arm were more likely to adopt contraception compared to women in the comparison arm (Sitrin D et al. 2020).”

**Materials and Methods**

 6. The description of the qualitative methods is comprehensive and the framework much appreciated.  

*Response*: Thank you.

 7. As the results also report quantitative data (Table 2), please add information on how you collected the data and reflect this mixed-method approach in the title, abstract and manuscript.

*Response:* Thank you for your comment. Table 2 shows results of observations (n=180) of forms completed by health extension workers (HEWs). We reviewed the forms and noted how HEWs filled in the forms including its completeness.  This was program monitoring data from a tracking tool to show that HEWs were able to fill the forms.

**Results**

 8. See commentary above on Table 2, which comes as a quantitative surprise.

 Thanks you. Please look at the response for number 7.

**Discussion and Conclusion**

 9. Without data on effectiveness from the main study, the reader may find it difficult to support the following statement: “Quantitative data on PPFP uptake will be published separately. Nevertheless, we feel the qualitative data on the IMCH card are sufficient to recommend use of prompts to encourage systematic integration of PPFP counseling and services.”

*Response:* Thank you. We modified the statement as:

 “The effectiveness of the interventions on PPFP uptake published separately and revealed that postpartum women in the intervention arm were more likely to adopt contraception compared to women in the comparison arm (Sitrin D et al. 2020). Based on the results of the effectiveness and qualitative studies, the IMCH card is feasible to use and we recommend use of prompts to encourage systematic integration of PPFP counseling and services.”

 10. In relation to the previous comment, no doubt about the usefulness of reminders to systematically address and avoid missed opportunities in relation to PPFP (and other health areas). Could you provide more information and discussion on the time requirement and potential trade-offs for

*Response:* Thank you for your comment. It turned out that the modified IMCH card *saves *health extension workers time. The following is taken from the results section under the sub-title “How acceptable and feasible is the modified IMCH card?” : “Unlike tools used in the past, HEWs documented all interventions in the modified IMCH card using a code, so it did not take much time to write each of the services provided to clients.”

# Peer review: Reviewer response for version 1

**DOI:** 10.21956/gatesopenres.14207.r28454

**Published:** 2020-01-31

**Authors:** Alemayehu Hailu

**Affiliations:** 1 Department of Preventive Medicine, School of Public Health, Addis Ababa University, Addis Ababa, Ethiopia

Overall, this study is a very nice addition to the literature on postpartum family planning counseling. The results presented adds new knowledge about PPFM counseling.

 In this paper, the authors try to present findings on how a section guide to counselling of mothers on post-partum family planning can be integrated into the main/traditional IMCH follow-up card. They qualitatively explore the feasibility of the integration of the PPFM part to the main IMCH and examine the acceptability by HEWs. This study is also part of a large quasi-experimental study.

 There are two points where a further description is needed. First, what is the current arrangement in the Ethiopian health system to revise, add a new section, remove, or change a section into the current way of doing things (i.e., in this case, the follow-up card)? A description of the current possible mechanism to revise the IMCH card (in methods/discussion section) would help readers.  

 Second, in your conclusion section, you have mentioned that ‘The Ethiopian FMOH plans to scale a modified IMCH card’. However, recently the Ethiopian Ministry of Health is also initiating an electronic-based medical recording system at all levels, and for the primary level reproductive health services at health posts and health centers in particular. Would you please describe (in your discussion) how these paper-based PPFM counselling guide can fit into the ministries future direction.

Is the work clearly and accurately presented and does it cite the current literature?

Yes

If applicable, is the statistical analysis and its interpretation appropriate?

Not applicable

Are all the source data underlying the results available to ensure full reproducibility?

Yes

Is the study design appropriate and is the work technically sound?

Yes

Are the conclusions drawn adequately supported by the results?

Yes

Are sufficient details of methods and analysis provided to allow replication by others?

Yes

Reviewer Expertise:

Health Economist, Health systems research, Health policy, Public health, Health benefits package design.

I confirm that I have read this submission and believe that I have an appropriate level of expertise to confirm that it is of an acceptable scientific standard, however I have significant reservations, as outlined above.

# Response

**Published:** 2021-04-04

**Authors:** Muluneh Yigzaw Mossie

Overall, this study is a very nice addition to the literature on postpartum family planning counseling. The results presented adds new knowledge about PPFM counseling.

 In this paper, the authors try to present findings on how a section guide to counselling of mothers on post-partum family planning can be integrated into the main/traditional IMCH follow-up card. They qualitatively explore the feasibility of the integration of the PPFM part to the main IMCH and examine the acceptability by HEWs. This study is also part of a large quasi-experimental study.

 There are two points where a further description is needed. First, what is the current arrangement in the Ethiopian health system to revise, add a new section, remove, or change a section into the current way of doing things (i.e., in this case, the follow-up card)? A description of the current possible mechanism to revise the IMCH card (in methods/discussion section) would help readers.  

*Response:* Thank you for your comment. We think that the following description at the 5 ^th^ paragraph of the discussion section (page 17) addresses your concern.

 “Based on preliminary experience from this study, the FMOH approved revisions to include PPFP counseling, method choice, and uptake in the pregnancy, postnatal, and immunization sections of the new IMCH card. However, the study’s innovation of prompting HEWs to assess various LAM criteria, re-labeled as pregnancy risk, was not incorporated into revisions to national tools. HEWs expressed appreciation for this component as it introduced “a new way of counseling” women on the possibility of a pregnancy. This is encouraging for resolving the persistent problem of timely transition from LAM to other modern contraception in programs that have sought to promote LAM (Cooper et al., 2014; Cooper et al., 2016; Kouyaté et al., 2015). For Ethiopia, additional changes to the IMCH cards are unlikely in the short term given the recent revisions of national tools. In the short term, prototyping an alternative means to arrive at the same result, such as separate job aids, or stickers to add to the IMCH card or folder, can be explored”.

 Second, in your conclusion section, you have mentioned that ‘The Ethiopian FMOH plans to scale a modified IMCH card’. However, recently the Ethiopian Ministry of Health is also initiating an electronic-based medical recording system at all levels, and for the primary level reproductive health services at health posts and health centers in particular. Would you please describe (in your discussion) how these paper-based PPFM counselling guide can fit into the ministries future direction.

*Response:* Thank you for your comment. Perhaps this comment was about the electronic community health information systems and DHIS2. Regarding the digitalization of records, we point out the following in the last paragraph of the discussion section:

  “Our study sought to track PPFP through the continuum of care using paper-based records. Transforming such paper-based records to digital health records has potential to further improve service integration. Exploring such applications can incorporate items related to PPFP decision-making and follow up for timely adoption of a contraceptive prior to return of postpartum fecundity”.
